# Morphological and palaeoecological aspects of fossil insects unveiled by UV-A light

**DOI:** 10.1016/j.mex.2024.102794

**Published:** 2024-06-19

**Authors:** Mathieu Boderau, Corentin Jouault, Camille Aracheloff, Valérie Ngô-Muller, Michael S. Engel, Serge Berthier, Bernd Schöllhorn, Diying Huang, André Nel, Romain Garrouste

**Affiliations:** a Institut de Systématique, Évolution, Biodiversité (UMR 7205), MNHN, CNRS, SU, EPHE-PSL, UA, CP50, 57 Rue Cuvier, F-75005 Paris, France; b Géosciences Rennes (UMR 6118), Univ. Rennes, CNRS, F-35000 Rennes, France; c Institut des Sciences de l’Évolution de Montpellier (UMR 5554), Université de Montpellier, CNRS, Place Eugène Bataillon, F-34095 Montpellier, France; d Paris-Cité University, Life Sciences Department, 5, rue Thomas Mann, F-75013 Paris, France; e American Museum of Natural History, Central Park West at 79th Street, New York, NY 10024-5192, USA; f Facultad de Ciencias Biológicas, Universidad Nacional Mayor de San Marcos, Lima, Peru; g Departamento de Entomología, Museo de Historia Natural, Universidad Nacional Mayor de San Marcos, Avenida Antonio Álvarez de Arenales 1256 Jesús María, Lima 14, Peru; h Institut des Nanosciences de Paris (UMR 7588), CNRS, Sorbonne University, F-75005 Paris, France; i Université Paris Cité, Laboratoire d'Electrochimie Moléculaire, F-75013 Paris, France; j Key Laboratory of Palaeobiology and Petroleum Stratigraphy, Nanjing Institute of Geology and Palaeontology, Chinese Academy of Sciences, Nanjing 210008, China

**Keywords:** UV-light induced fluorescence observation of insect compression fossils, UV-light imaging, Taxonomy, Palaeontomology, Palaeoecology, Fluorescence

## Abstract

Studying insect fossils, particularly those preserved as compressions in sedimentary matrices, can be difficult due to the taphonomic processes that often result to poor preservation and contrast of structures compared to the embedding matrix. To address this, we propose a user-friendly and simple methodology based on UV-light to study insect fossils and select specimens of interest for more advanced imagery exploration. While UV-light imaging has been previously applied to compressions of arthropod fossils, it typically involved laser light sources. Our approach allows the investigation of fossils using an affordable, compact, and portable UV-light source, along with a simple and replicable low-cost protocol.

• The methodology is based on UV-light induced natural fluorescence of sediment and fossil remains.
• UV-light is effective on compression fossils to gain natural contrast and enhance observation of body structures like veins or setae on wings.
• UV-light is effective to reveal palaeoecological information such as pollen grains preserved on specimens, especially near or on putative pollinator or pollen-eating taxa.

## Graphical abstract

**Figure:**
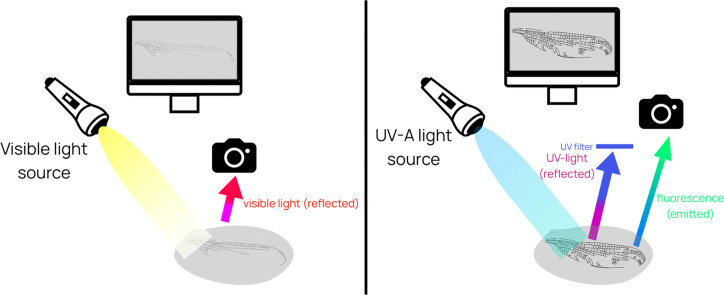

Specifications table

**Table:** 

Subject area:	Earth and Planetary Sciences
More specific subject area:	Palaeontology
Name of your method:	UV-light induced fluorescence observation of insect compression fossils
Name and reference of original method:	J.T. Haug, C. Haug, V. Kutschera, G. Mayer, A. Maas, S. Liebau, C. Castellani, U. Wolframn, E.N.K Clarkson, D. Waloszek. Autofluorescence imaging, an excellent tool for comparative morphology, Journal of Microscopy, 244 (2011): 259–272, doi: http://dx.doi.org/10.1111/j.1365–2818.2011.03534.x
Resource availability:	*n/a all resources are conventionally available.*

## Background

The taxonomic and paleoecological study of fossilized insect remains can be complicated due to taphonomic processes, which often distort or fragment key characters, or sometimes make them indistinguishable from the surrounding matrix. This is especially the case with those preserved as compressions or impressions in sedimentary rocks. The challenge for palaeoentomologists is to gather and observe as many characters as possible amidst these limitations. Achieving this goal necessitates the utilization of complementary imagery methods, leveraging sophisticated tools to ensure the most accurate interpretations are made.

Traditional approaches in palaeontomology rely on optical observation through a stereomicroscope or binocular magnifier. However, recent advancements have introduced a variety of news tools based on different sources of the electromagnetic spectrum. X-ray computed micro-tomography (µ-CT Scan) is frequently used to investigate insect fossils in amber [[1], [2], [3], [4]] and even for studying exceptionally preserved compression fossils [5]. Another non-destructive technique, Confocal Laser Scanning Microscopy (CLSM), is often used to study insect fossils in amber, exploiting the autofluorescence of the arthropod exoskeleton [6,7]. This autofluorescence property of chitinous exoskeletons extends to extant bugs (Hemiptera) and their compression fossils [8]. It facilitates, for example, the identification of pollen preserved on insect legs [9,10].

While all of these techniques are useful for palaeoentomologists in taxonomic description and ecological reconstruction, they are time-consuming and demand expensive equipment. Here, we propose a new methodology for enhancing observation with a simple, portable, and economical device based on the detection of “autofluorescence” or natural fluorescence as it has been performed for non-insect arthropods, vertebrates, or molluscs [[11], [12], [13]].

We demonstrate here that this simple method enhances and improves morphological interpretations and reveals palaeoecological information. Furthermore, we highlight its utility as a highly effective means for field- or museum-based screening and selection of specimens for further physicochemical analysis and imaging using more sophisticated techniques (e.g., X-ray fluorescence, synchrotron-radiation tomography) [14].

### Method details

**Fig. 1:**
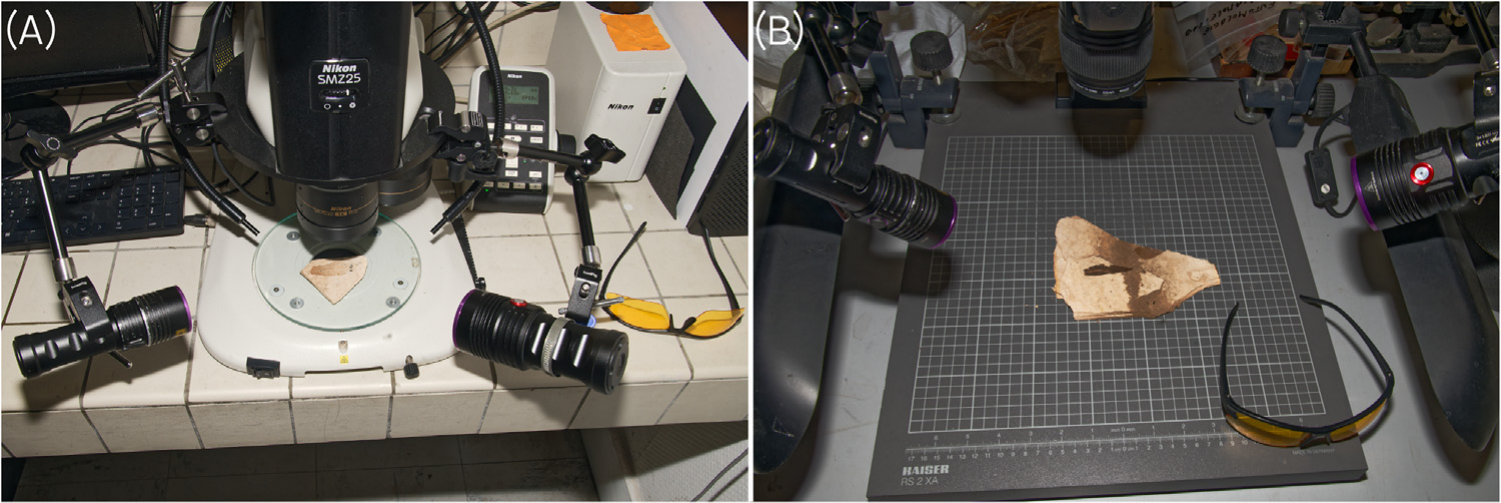
Setup designed for UV-light photography of insect fossils for (A) a stereomicroscope and (B) a statif rail.

**Equipment and setup for simple UV-light observation of insect fossils.** The equipment needed for examining fossil insects under UV light is simple: at least a compact, affordable, portable lamp emitting UV-A light at 370 nm; a pair of UV-light protective safety glasses; a photographic setup combining either a camera mounted on a statif rail or a stereomicroscope, macro lenses equipped with UV filters; a software for photo editing (Fig. 1).

**Maximum wavelength of the UV-A light source**. In this study, a commercial LED flashlight, equipped with an adapted band pass filter, was used as the UV-A light source. A maximum wavelength (λ_max_) of 365 nm was indicated by the vendor. However, upon measuring its emission spectrum with a calibrated Avantes AVASpec 2048SL spectrophotometer, it was necessary to correct the value of λ_max_ to 371 nm (Figure S-1, suppl. inf.). This corrected maximum wavelength falls within the UV-A range of the electromagnetic spectrum, and significantly differs from the other light sources typically utilized in other invertebrate palaeontological studies. In contrast, those studies commonly employ lasers with maximum wavelengths in the visible blue range [6,13].

**Natural fluorescence**. The methodology is based on the concept of natural fluorescence, often referred to as “autofluorescence” or “epifluorescence” in the context of microscopic techniques. This optical intrinsic property is inherent to various natural materials such as minerals or organic and inorganic bio-matter [15]. Fluorescence, the most common type of photo-luminescence, is defined as the spontaneous light emission upon photo-excitation. When a fossil is illuminated with light of a specific wavelength, it emits light of a different wavelength, which possesses less energy than the initial excitation wavelength. In our methodology, we employe an excitation wavelength of 371 nm, falling within the UV-A range. This contrasts with conventional visible light sources, such as violet or blue, which have historically been developed for cellular and molecular biology fluorescence imaging.

**Photographic settings**. The accurately detected fluorescence emission, it is imperative to minimize interference from ambient light. When dealing with UV-induced visible fluorescence, it's essential to position the setup accordingly in a dark room (e.g., in a basement). Following this, the ISO setting controlling the sensitivity of the camera sensor (sensitivity of the sensor) must be increased in order to gather sufficient information and photon capture.

**Taxonomic sampling**. We demonstrate the effectiveness of our methodology through case studies (published or in preparation), featuring fossils from different outcrops, taphonomic conditions, and from a different insect orders, allowing us to assess the success of the methodology regardless of deposit or taxonomic affiliation.

### ***Method validation***

**Fig. 2:**
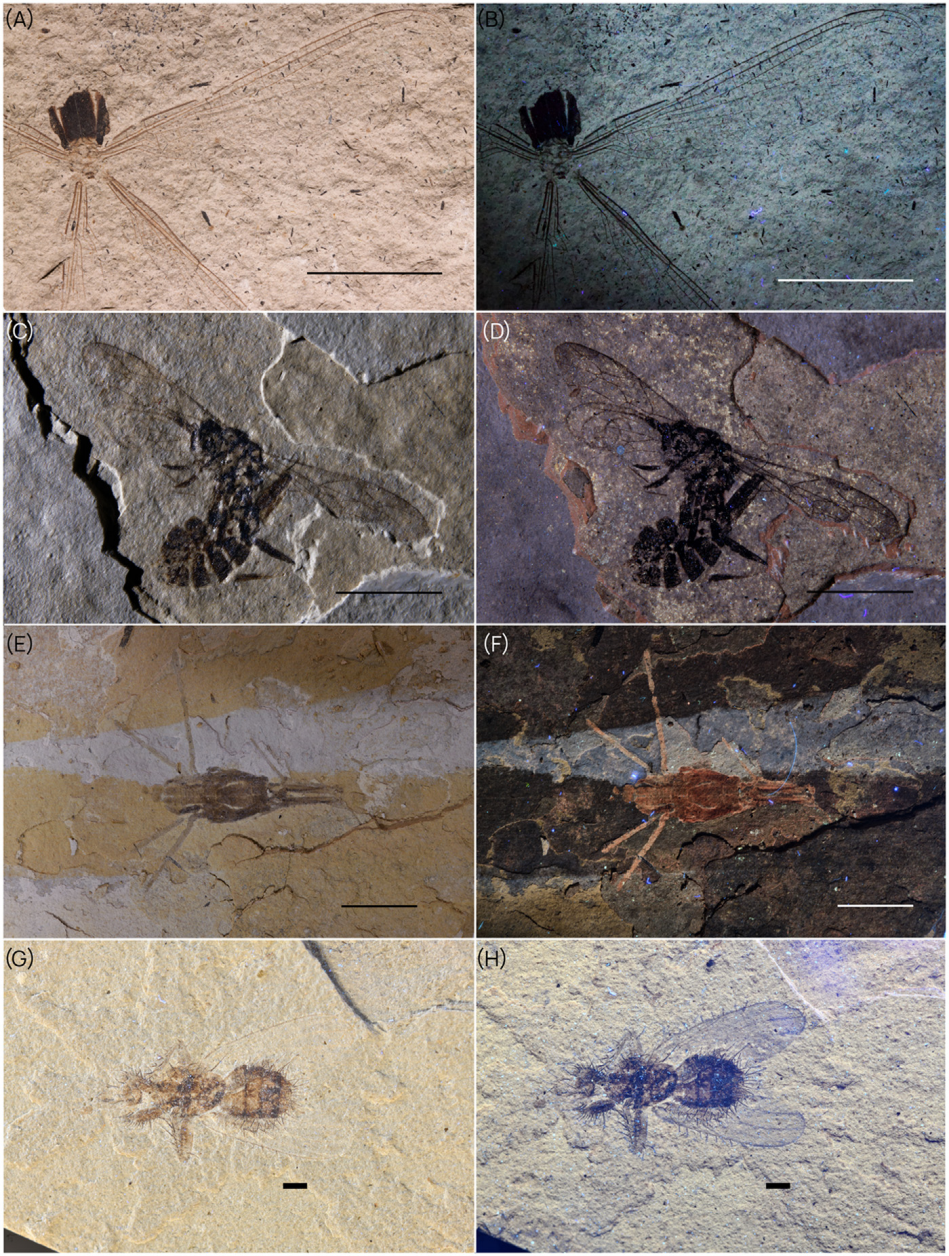
Differential contrast between normal light (A,C,E,G) and UV-light photographs (B,D,F,H) for different insect compression fossils. A-B: *Stenolestes oeningensis* (Odonata: Sieblosiidae) holotype Pt. I. 664, scale bars = 1 cm. C-D: *Lithoserix oublieri* Viertler 2024 (Hymenoptera: Ichneumonidae) holotype PNRL-SIG-216, scale bars = 5 mm. E-F: *Oligoptilomera luberonensis* (Hemiptera: Gerridae) Nel et al. 2023 holotype PNRL 2715, scale bars = 5 mm. G-H: *Eornithoica grimaldii* (Diptera: Hippoboscidae) Nel et al. 2023 holotype FOBU17740, scale bars = 1 mm.

**Fig. 3:**
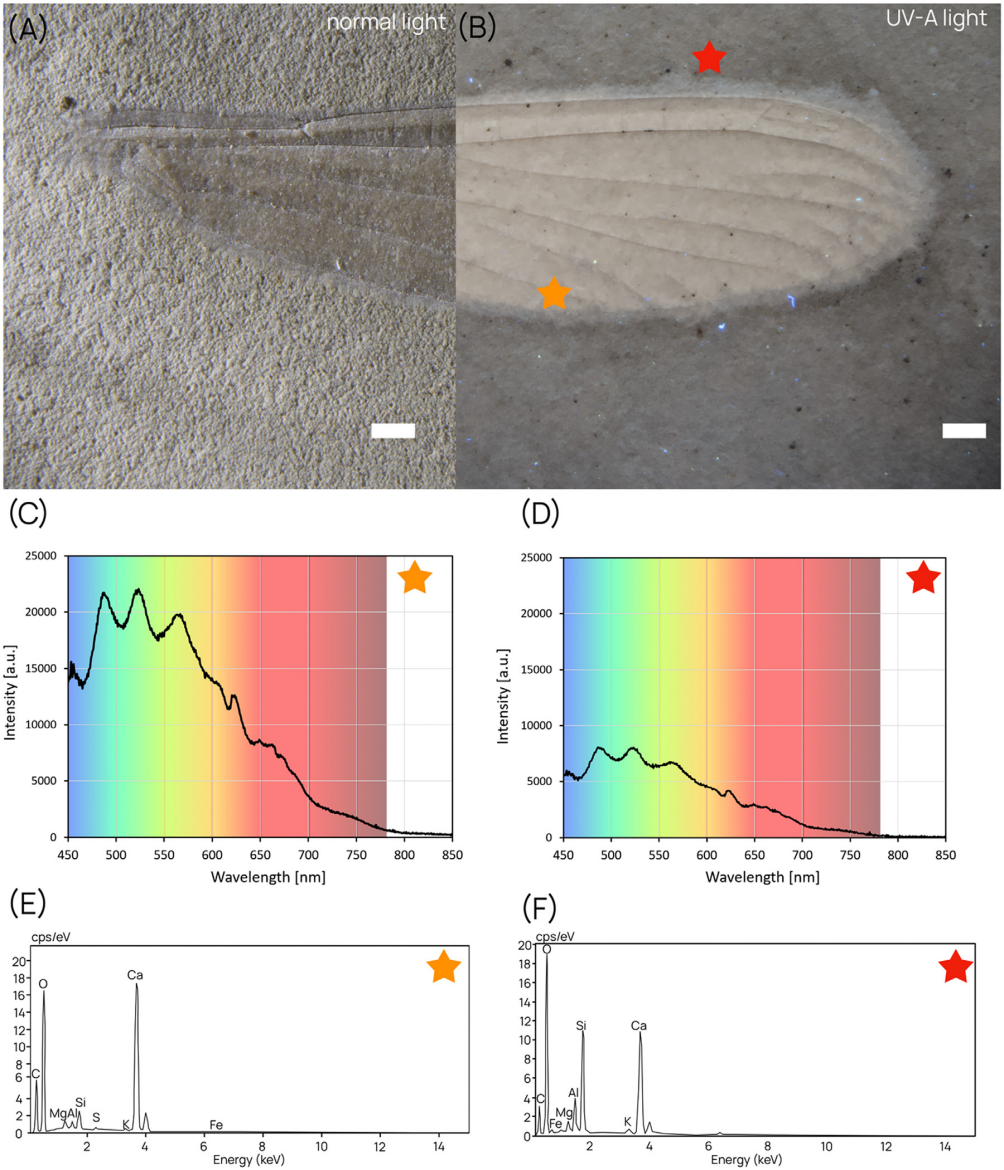
Differential chemical signals in a fossil damselfly (Odonata: Zygoptera) from the Oligocene Aix outcrop. (A-B): Photographs of the specimen under normal light (A) and UV-A light (B), with colored stars indicating the sample points for emission spectrum measurements and chemical analysis, scale bars = 1 mm. (C-D): UV-A (371 nm) induced fluorescence emission spectrum of the fossil (C) and the sedimentary matrix (D). (E-F): EDS elemental spectrum of the fossil (E) and of the sedimentary matrix (F). Abbreviation: cps = counts per second.

**Accuracy of morphological interpretations under normal light vs. UV-light**. From the diversity of investigated specimens, natural fluorescence significantly enhances the contrast between the sediment and the structures of the fossilized organic matter (Fig. 3). This contrast enhanced photographic acquisition and facilitated, for instance, the identification of wing veins, which are often challenging to observe under natural light and are among the most important features to ascertain taxonomic attribution (Fig. 2.A-D). These case studies also demonstrated that UV-light-induced fluorescence accentuates exceptionally fine characters such as the setae covering the body of the fly *Eornithoica grimaldii* (Fig. 2.G-H, [18]).

### Physico-chemical analysis of fossils and sediment

To confirm whether the contrast obtained under UV illumination is linked to the difference in chemical composition between the fossil and its sedimentary matrix, we first recorded fluorescence emission spectra with an Avantes AVASpec 2048SL spectrophotometer under UV-A light (371 nm). Subsequently, we conducted a chemical analysis by energy dispersive spectroscopy (EDS). This analysis was performed using a MEB FEG TESCAN CLARA in dual EDS mode, under variable pressure mode, enabling observation of non-conductive and non-metallized samples. This methods allowed to produce secondary electron/back scattered electron (SE/BSE) imaging, fast element analysis, and hyperspectral chemical mapping on a fossil of Zygoptera (damselfly) from the Oligocene Aix outcrop. Top compare the chemical signal, measurements were taken both ‘within’ the fossil and outside in the sedimentary matrix (Fig. 3.A-B). The results from the chemical analysis were then visualized in EDS elemental spectrum (Fig. 3.E-F).

Observation of the specimen under UV-A-light-induced fluorescence provided a considerable gain in contrast, allowing for detailed observation of the body characters (Fig. 3.A vs. B). This contrast enhancement is evident not only in the photographs but also in the emission spectra of both the fossil and its matrix (Fig. 3.C,D).

**Fig. 4:**
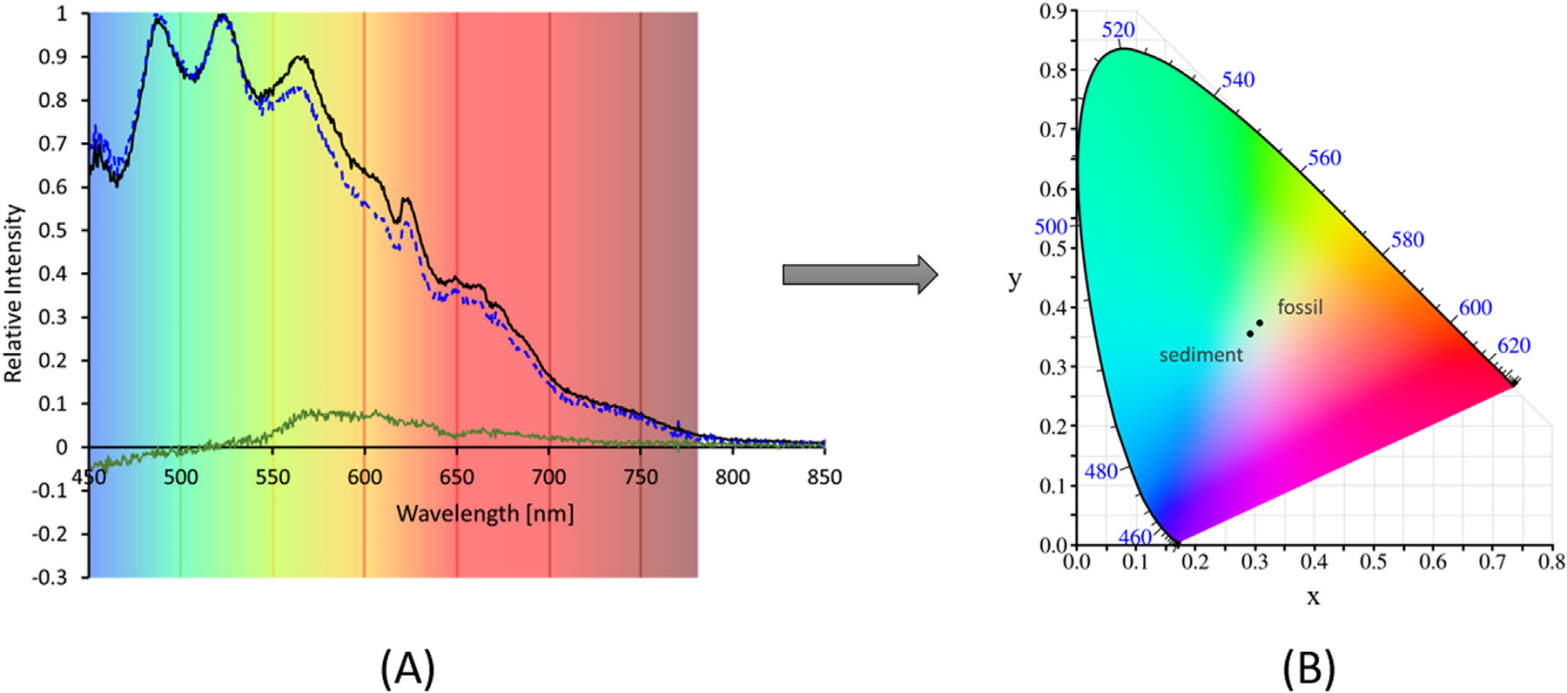
(A) Normalized fluorescence emission spectra of the fossil (black trace) and the surrounding sediment (broken blue trace) upon UV-A excitation (λ_max_ = 371 nm) with a LED flashlight. Corresponding differential spectrum (green trace). (B) The respective calculated colorimetric codes corresponding to the normalized spectra, and represented in the CIE 1931 system.

The similarities in the shape of the spectra corresponding to the fossil (Fig. 3.C) and its matrix (Fig. 3.D) are likely due to a similar chemical composition. However, the fluorescence intensity is significantly higher in the fossil, although quantification of this difference is not feasible with the present experimental setup. To address this, both emission spectra were normalized (Fig. 4.A), revealing a disparity in their shape, which is clearly also expressed in the corresponding differential spectrum. Converting the normalized spectra into color codes of the colorimetric system CIE 1931, adapted to human vision sensitivity, yielded two distinct and significantly separated colors, both close to the white origin (Fig. 4.B): fossil (*x* = 0.310483694, *y* = 0.370985016) and sediment (*x* = 0.294099197, *y* = 0.352832879).

We assume that this difference in emission, which translates to a difference in contrast, is linked to the different chemical composition of the fossil and the surrounding matrix. Indeed, based on the EDS analysis (Fig. 3.E,F), the matrix is highly enriched in silicon compared to the fossil, which is enriched in calcium. The presence of high levels of silicon in the sedimentary matrix of the Oligocene-aged Aix outcrop is congruent with the results of Olcott et al. (2022), and the abundance of diatoms, while the high levels of calcium and oxygen in the fossil indicate a carbonate composition.

**Comparison between different light sources**. We conducted a comparative analysis to assess the influence of light sources on the quality of observation with photographs. For this study, we selected a fossil cricket (Grylloidea sp.) from the Jurassic of Lias (see Table 1). High-resolution photographs of the specimen were captured under normal light, and these were compared with photographs captured under two alternative light sources emitting at higher frequencies: 371 nm (UV-A) and 395 nm (violet). A Nikon Z7 II attached to a Nikon SMZ25 stereomicroscope was used to photograph the specimen under each source light to evaluate the comparative effects on observation quality. EDS-X imaging was used (C-element map) for this specimen.

**Table 1:** Taxonomic sampling.

Taxonomy	Outcrop	Age (Mya)	Collection	Reference
*Oligoptilomera luberonensis* (Hemiptera: Gerridae)	Murs (France)	33.9–28.4 (Oligocene)	Parc Naturel Régional du Luberon	[16]
*Stenolestes oeningensis* (Odonata: Sieblosiidae)	Öhningen (Germany)	12.75–11.00 (Miocene)	Paleozoological Collection ETH Zürich (Switzerland)	[17]; Boderau unpubl. data
‘*Apis*’ *adamatica* (Hymenoptera: Megachilidae)	Öhningen (Germany)	12.75–11.00 (Miocene)	Paleozoological Collection ETH Zürich (Switzerland)	[17]; Engel unpubl. data
Grylloidea sp. (Orthoptera)	Lias (France)	201.3–196.5 (Jurassique)	Muséum national d'Histoire naturelle	Unpublished fossil
*Eornithoica grimaldii* (Diptera: Hippoboscidae)	Green River (USA)	56.0–47.8 (Eocene)	Fossil Butte National Monument, Wyoming, USA	[18]
*Lithoserix oublierus* (Hymenoptera: Ichneumonoidea)	Campagne-Calavon Formation (France)	31.0–30.0 (Oligocene)	Parc Naturel Régional du Luberon	[19]

**Fig. 5:**
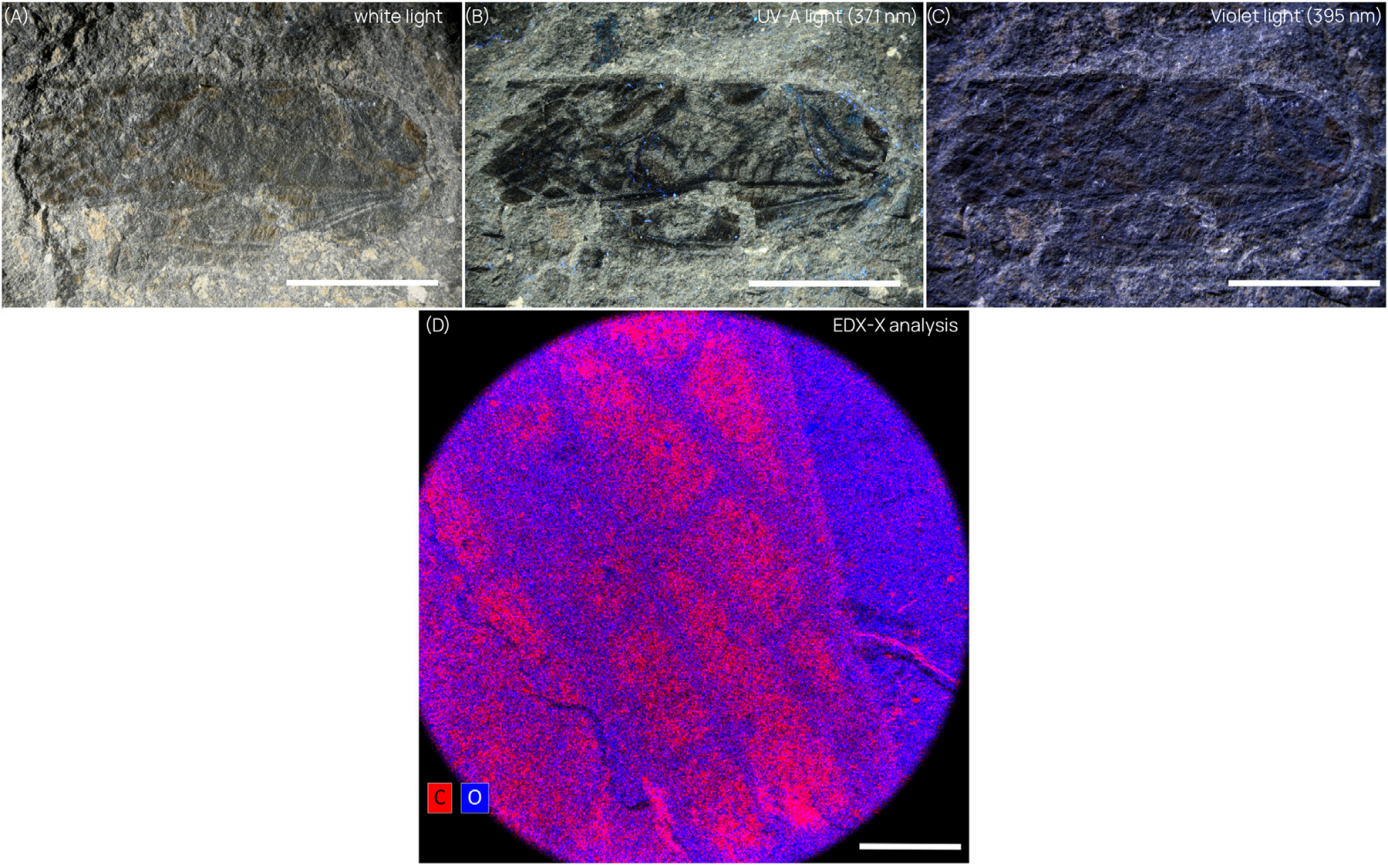
Specimen of Grylloidea sp. (Orthoptera) MNHN-FA57514 under (A) specimen under normal light (white). (B) UV-A light (371 nm) and (C) violet light (395 nm). (D) Element mapping for C (carbon) and O (oxygen), EDX-X analysis of the margin of the wing. Scale bars: A-C = 1 cm. *D* = 500 µm.

Under normal light conditions (Fig. 5.A), it is difficult to identify the forewing venation and its color pattern. In comparison, under UV-A light (Fig. 5.B), a gain in contrast is evident, rendering it much easier to distinguish the veins composing the forewing and the traces of coloration. When using a violet lamp emitting light at a maximum wavelength closer to visible blue (395 nm, Fig. 5.C), the resulting image resembles that obtained with normal white light, with the forewing veins once again proving difficult to distinguish. This difference in contrast between the two wavelengths could be explained by a weaker fluorescence intensity and the presence of excessive visible light in the 395 nm wavelength, owing to the absence of a band pass filter in the commercially available devices. This excess of visible light saturates the autofluorescence of the sediment, thereby diminishing the contrast between the fossil and the surrounding matrix.

The mapping (Fig. 5.D) is representative of this kind of taphonomy observed in fossil insects from the Jurassic outcrop of Lias, wherein the compression fossil is preserved with organic matter remaining in a thin layer superimposed on the sedimentary matrix. This matrix is more fluorescent than the fossil itself, with no apparent mineralized diagenesis. However, it is important to note that the method is not relevant for analyzing rare elements, which necessitate higher-tech methods such as X-ray fluorescence, especially with synchrotron-radiation excitation.

**Fig. 6:**
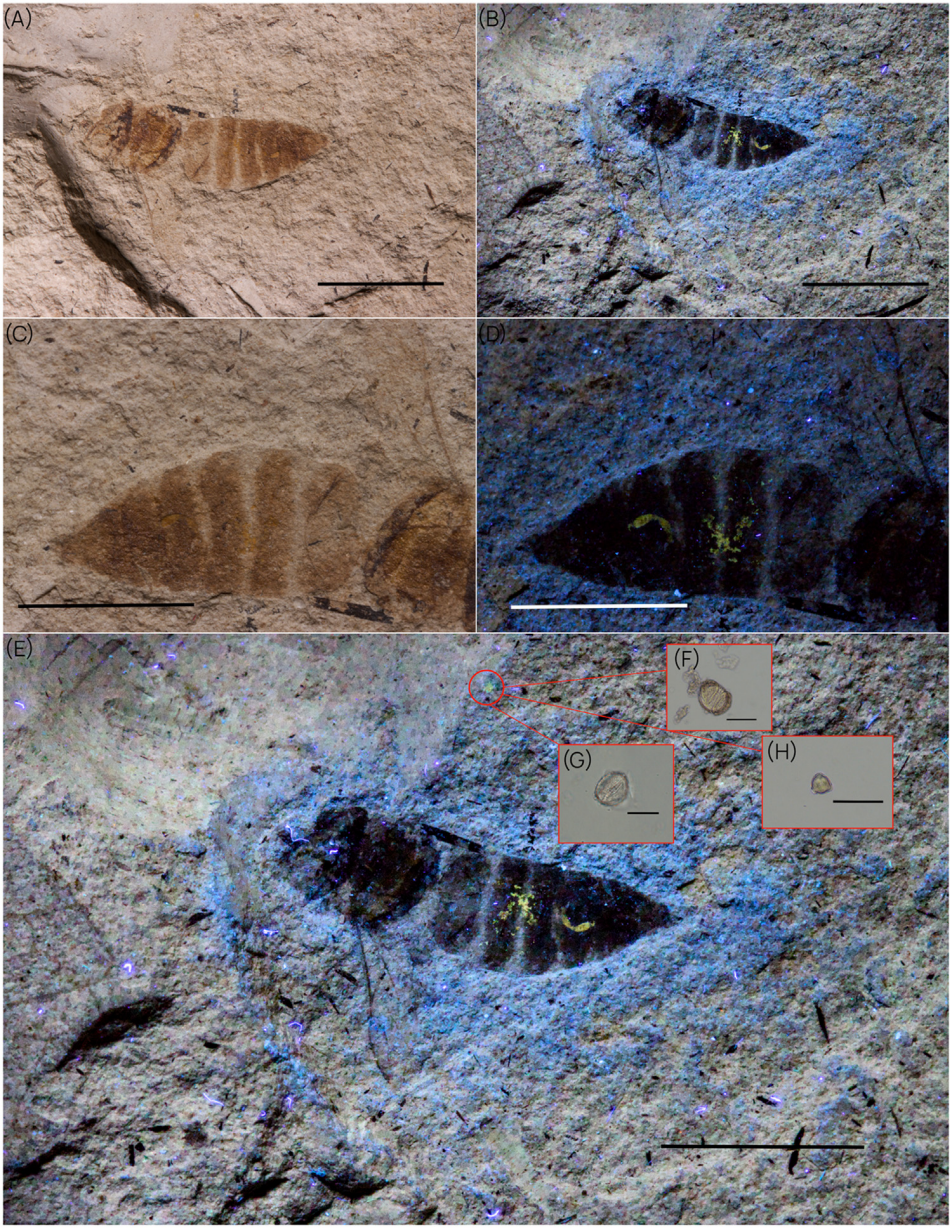
‘*Apis*’ *adamatica* (Hymenoptera: Megachilidae). (A) General habitus of specimen under normal light. (B) habitus under UV-light 371 nm. (C) Abdomen of insect under normal light. (D) abdomen under UV-light 371 nm. (E) General habitus under UV-light 371 nm, with the red circle indicating the locality of pollen grains. (F) *Pinus sp*. pollen grain. (G-H) Two pollen grains of monocotyledonous plants. Scale bars : A-*E* = 5 mm; *G* = 50 µm; *H* = 20 µm and *I* = 50 µm.

**Application of the methodology for palaeoecology: identification of pollen grains using UV-light**. We investigated the holotype ETH 8855 of ‘*Apis*’ *adamatica* (Hymenoptera: Megachilidae) (unpubl. data) from the ETH Zürich collection of Miocene arthropods from Öhningen, Germany (Fig. 6). Under UV-light, fluorescent yellow spots on the abdomen of the specimen (Fig. 6.B) are immediately evident, as well as some located in the surrounding matrix (Fig. 6.E), which were challenging to discern under normal light (Fig. 6.C). We sampled the spots in the matrix and observed them under photonic microscope – the spots on the insect were not sampled to avoid damaging the unique and fragile holotype. Analysis of this material revealed the presence of pollen grains of three different morphologies, representing two different monocotyledonous pollen forms (Fig. 6.H-I) and one of pollen from *Pinus* sp. (Fig. 6.G-I).

**Conclusion**. This article formalizes a methodology demonstrated through several case studies leveraging natural fluorescence to improve the observation of key characters on insect fossils. This new method significantly enhances the observation of morphological features critical for taxonomic attribution, such as wing venation, and also facilitates the detection of palaeoecological associations preserved alongside the fossil, such as pollen grains. Through our examples, we empirically illustrate the versatility of this methodology, demonstrating its applicability across various insect lineages and deposits of varying compositions and ages.

We demonstrate that the contrast gained under UV-light is primarily linked to the fluorescence of the sedimentary matrix rather than the transformed remains of the insect. We emphasize that irradiation of fossils with UV-A light improves the quality of observation due to the emitted fluorescence, contrasting with traditional sources using blue light.

While the principle of using natural fluorescence in palaeontology is widespread in different communities [6,[11], [12], [13]] employing multispectral devices or fluorescence microscopes can be prohibitively expensive. Herein, we demonstrated that compact, affordable devices can yield significant imaging results, adaptable to specimen of any size using standard imaging systems (CMOS sensors of the majority of cameras).

We claim numerous advantages with this methodology compared to current trends in palaeoentomology. The contrast obtained with a UV-light lamp streamlines the techniques needed to achieve similar results, such as reflectance-transformation imaging, which can be complex and more expensive. Additionally, it circumvents the need for alcohol application to enhance specimen contrast, which can potentially damage unique fossils, particularly over time and repeated applications. Importantly, this methodology is a new way to gain contrast without forcibly and artificially editing images, preserving the integrity of morphological structures. Its simplicity and affordability make it a valuable asset for screening fossil insect collections and unveiling previously overlooked features.

Furthermore, the fluorescence appears to correlates with the composition of the sediment, suggestion potential for investigating the link between fluorescence and sediment chemical composition.

This method offers numerous advantages to enhance the information garnered from fossil specimens. It sheds light on the morphology of fossils and elements related to their composition, providing insights into the sedimentology diagenesis of fossils, thus aiding taphonomic studies. Finally, it opens avenues for paleoecological inferences concerning the depositional environment and the preservation of biotic associations, such as the otherwise difficult localization of pollen grains on or within fossil insects.

## Limitations

None.

## CRediT authorship contribution statement

**Mathieu Boderau:** Methodology, Investigation, Conceptualization, Data curation, Writing – original draft, Writing – review & editing. **Corentin Jouault:** Methodology, Investigation, Data curation, Writing – review & editing. **Camille Aracheloff:** . **Valérie Ngô-Muller:** Writing – review & editing. **Michael S. Engel:** Investigation, Data curation, Writing – original draft, Writing – review & editing. **Serge Berthier:** Investigation, Data curation, Writing – review & editing. **Bernd Schöllhorn:** Investigation, Data curation, Writing – review & editing. **Diying Huang:** Investigation, Data curation, Writing – review & editing. **André Nel:** Conceptualization, Data curation, Writing – original draft, Writing – review & editing. **Romain Garrouste:** Methodology, Investigation, Conceptualization, Data curation, Writing – original draft, Writing – review & editing, Supervision.

## Declaration of competing interest

The authors declare there is no conflict of interests.

## Data Availability

No data was used for the research described in the article.

## Appendix Supplementary materials

Image, application 1
