# Supplementary figures and images for: Morphological and palaeoecological aspects of fossil insects unveiled by UV-A light
Source: MethodsX. 2024 Jun 19;13:102794. doi: 10.1016/j.mex.2024.102794 (PMC11259923; doi:10.1016/j.mex.2024.102794)

**Supplementary figures**

**Figure S1.** Emission spectrum of the employed UV-A light.
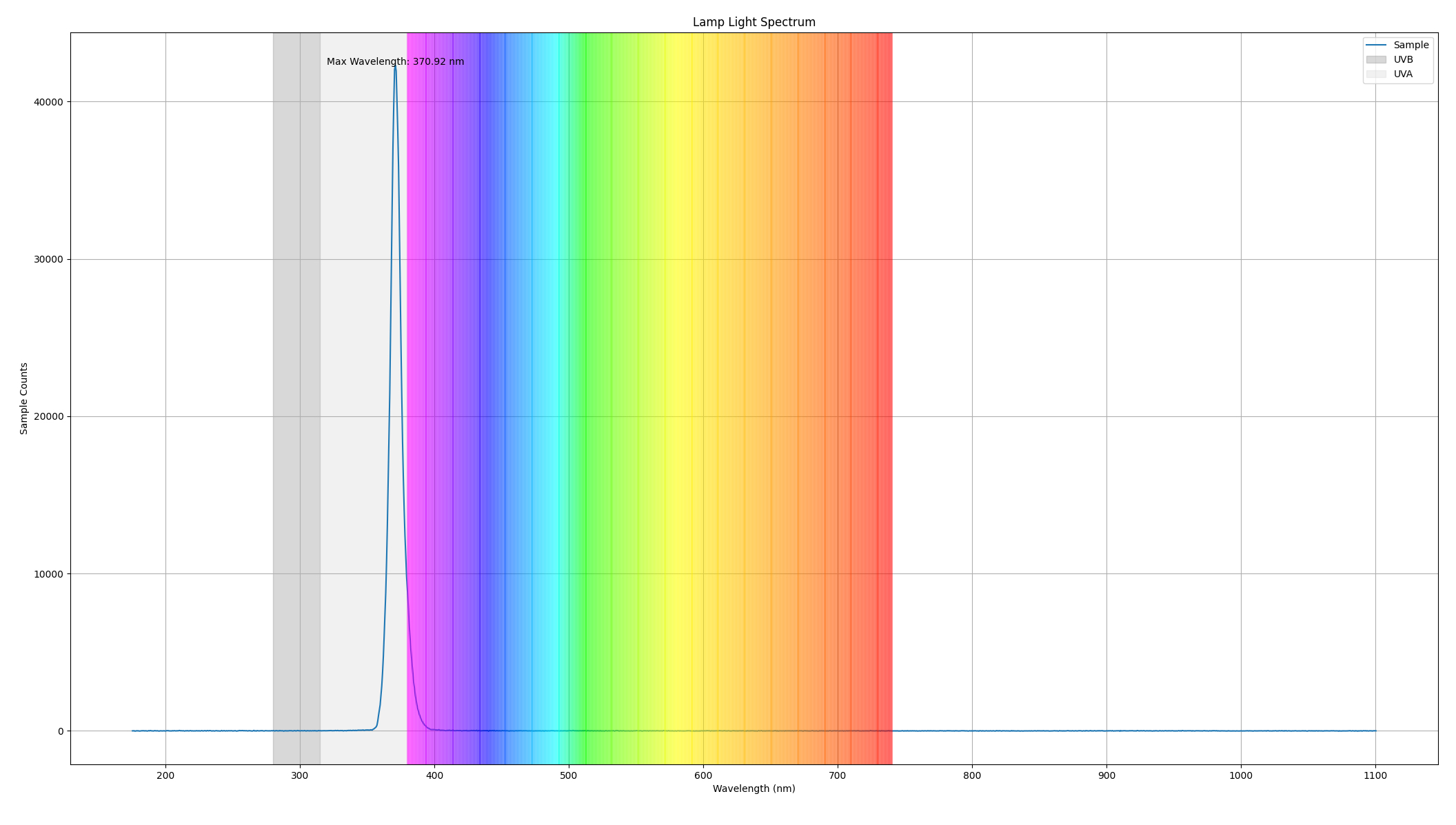

Supplement: Supplementary file 1 [file mmc1.docx]
